# The Signaling Pathways, and Therapeutic Targets of Antiviral Agents: Focusing on the Antiviral Approaches and Clinical Perspectives of Anthocyanins in the Management of Viral Diseases

**DOI:** 10.3389/fphar.2019.01207

**Published:** 2019-11-08

**Authors:** Pardis Mohammadi Pour, Sajad Fakhri, Sedigheh Asgary, Mohammad Hosein Farzaei, Javier Echeverría

**Affiliations:** ^1^Department of Pharmacognosy, School of Pharmacy and Pharmaceutical Sciences, Isfahan University of Medical Sciences, Isfahan, Iran; ^2^Pharmaceutical Sciences Research Center, Health Institute, Kermanshah University of Medical Sciences, Kermanshah, Iran; ^3^Isfahan Cardiovascular Research Center, Cardiovascular Research Institute, Isfahan University of Medical Sciences, Isfahan, Iran; ^4^Departamento de Ciencias del Ambiente, Facultad de Química y Biología, Universidad de Santiago de Chile, Santiago, Chile

**Keywords:** anthocyanins, natural compounds, antiviral, viral diseases, signaling pathways, therapeutic targets

## Abstract

As the leading cause of death worldwide, viruses significantly affect global health. Despite the rapid progress in human healthcare, there are few viricidal and antiviral therapies that are efficient enough. The rapid emergence of resistance, and high costs, as well as the related side effects of synthetic antiviral drugs, raise the need to identify novel, effective, and safe alternatives against viral diseases. Nature has been of the most exceptional help and source of inspiration for developing novel multi-target antiviral compounds, affecting several steps of the viral life cycle and host proteins. For that matter and due to safety and efficacy limitations, as well as high resistance rate of conventional therapies, hundreds of natural molecules are preferred over the synthetic drugs. Besides, natural antiviral agents have shown acceptable antiviral value in both preclinical and clinical trials.This is the first review regarding molecular and cellular pathways of the virus life cycle, treatment strategies, and therapeutic targets of several viral diseases with a particular focus on anthocyanins as promising natural compounds for significant antiviral enhancements. Clinical applications and the need to develop nano-formulation of anthocyanins in drug delivery systems are also considered.

## Introduction

As unique obligate pathogens mostly dependent on live organisms, viruses are becoming a leading cause of death in life-threatening diseases (Becker et al., 2006). Due to their simplest form of an encapsidated nucleic acid and their ability to borrow molecular equipment from host cells to complete their replication cycle, viruses have a few targets for antiviral agents (Schneider et al., 2014).

In spite of tremendous advancements in antiviral drugs, there are several problems with the existing antiviral treatments, including safety and efficacy limitations, as well as their high costs (Perrin and Telenti, 1998; Revuelta-Herrero et al., 2018). The catastrophic rise in viral infections and related mortality urged a growing need to provide safe and effective drugs to treat viral diseases. It has yet remained a challenge due to the small number of targets in viruses, the rapid evolution of viral genes, the rapid emergence of drug-resistant pathways, and the appearance of new viral strains through mutations (Martins et al., 2016). Furthermore, considering the increasing threat of viral diseases, the emphasis is always on the need to identify suitable alternative therapies targeting different steps in the viral replication cycle (Perrin and Telenti, 1998; Gogineni et al., 2015). Recent developments in the investigation of novel cellular and molecular mechanisms of virus invasion and replication have provided alternative or additional innovative and effective therapeutic strategies. There are several natural compounds already with proven antiviral value in preventing and/or attenuating viral diseases or that are waiting to be evaluated for therapeutic applications. Anthocyanins are believed to display an array of beneficial actions on human health and well-being. Due to our increasing understanding and awareness of the potential beneficial human health effects, research on anthocyanins has recently intensified. A growing number of studies have recently shown the diverse beneficial effects of anthocyanins in vegetables and fruits, including the anti-cancer, anti-diabetes, ant-aging, anti-allergy, cardioprotection, anti-mutagenesis, and antimicrobial effects (Ghosh, 2005). Anthocyanins also constitute a flavonoids subfamily with neuroprotective, anti-inflammatory, and anti-oxidative properties (Amin et al., 2017). Besides, considering the inhibitory effects of anthocyanins on different pathways involved in virus life cycle, they would be hopeful antiviral therapies. Several other biological and pharmacological effects of anthocyanins are incessantly being investigated.

This is the first review regarding the current antiviral approaches and alternative, natural antiviral compounds while tackling particular attention to anthocyanins. We also focused our attention on the need to develop nanoformulation to improve the anthocyanin delivery system.

## Current Therapeutic Approaches for Viral Diseases and Their Restrictions

Antiviral drugs can be categorized into the inhibitors of fusion, uncoating, nucleic acid synthesis, integration, protease, and release (Pommier et al., 2005; Antonelli and Turriziani, 2012).

The interaction between the virus and the host cell membrane or receptor(s) is the first phase of the viral life cycle called fusion/entry. Fusion/entry inhibitors have been more provided for human immunodeficiency virus (HIV) treatment (Soriano et al., 2009; Tilton and Doms, 2010; Fumakia et al., 2016) and respiratory syncytial virus (RSV) prevention (Hu and Robinson, 2010; Lanari et al., 2010; Welliver, 2010). Targeting chemokine receptors (Kindberg et al., 2008; Lim and Murphy, 2011) and glycoprotein (GP)-receptor interactions (Antoine et al., 2013), as critical co-transporters of entry phase, are also of the most attractive candidates to inhibit viral entry/fusion. A novel lipopeptide known as myrcludex B also blocks taurocholate co-transporting polypeptide (NTCP); the co-receptor which is crucial for either hepatitis B virus (HBV) and hepatitis D virus (HDV) entry into hepatocytes (Uhl et al., 2014).

The low pH of the endosome activates M2 proton channels following virus entry to acidify the viral interior and weaken the electrostatic interaction to allow viral uncoating (Pinto et al., 1992). Uncoating inhibitors have been used more against the influenza virus (InfV) by inhibiting the function of the M2 ion channel (Davies et al., 1964; Martin and Heleniust, 1991). However, the emergence of resistance to these drugs has been detected, which raises concerns regarding their widespread use (Guan and Chen, 2005).

Following uncoating, nucleic acid synthesis is the third step of the viral life cycle, which is mediated by viral enzymes, including RNA polymerase, DNA polymerase, and reverse transcriptase. So, these enzymes have been considered as alternative targets in many viral infections such as HBV (De Clercq, 2015), herpes simplex virus-1/2 (HSV-1/2) and HIV-1. There are more targets and, also, more drugs against HIV in comparison to other viruses. Viral polymerase inhibitors are classified into nucleotide reverse transcriptase inhibitors (NtRTIs), nucleoside reverse transcriptase inhibitors (NRTIs), and non-nucleoside reverse transcriptase inhibitors (NNRTIs) (Miller and Miller, 1982; Elion, 1993; De Clercq, 2009). A serious problem with the use of a nucleic acid synthesis inhibitor is the frequently-developed drug-resistance (Montaner et al., 2010). Despite a rapid mutation for the first-generation NNRTIs (e.g., nevirapine, efavirenz, delavirdine) as the efficacy limitation agent (de Béthune, 2010), the second generation NNRTIs (e.g., etravirine, rilpivirine) possess a flexible structure and overcome common NNRTI resistance-associated mutations (Schiller and Youssef-Bessler, 2009; Adams et al., 2010). Altogether, most antiretroviral therapies did not eradicate the viral infection. Instead, they limited the immune recovery and their long-term use results in resistance and toxicity (Pantaleo, 1997; Pantaleo and Perrin, 1998; Perrin and Telenti, 1998; Telenti and Paolo Rizzardi, 2000). Therefore, new antiviral classes with new targets are urgently needed.

Inhibitors that specifically target integration have also been approved for the treatment of HIV infection. Since integrase is a necessary enzyme for the replication step involving the integration of host cell DNA to the viral genome, it has become a validated target for developing anti-HIV agents (Hazuda et al., 2000; Pommier et al., 2005; Antonelli and Turriziani, 2012). New integrase inhibitors targeting Gag, as an antiviral target, indicated auspicious effects both in preclinical and clinical trials (Min et al., 1999b; Pommier et al., 2005; Grinsztejn et al., 2007).

Viral proteases also signify an appropriate target for the development of novel antiviral agents against HIV and hepatitis C virus (HCV) infections (Eron Jr, 2000). Furthermore, the genome of HCV owns an open reading frame (ORF) encoding a single long polyprotein, which is processed either by virus-encoded proteases or host cellular peptidases (Vassilaki et al., 2008).

Nowadays, using a combination therapy of protease inhibitors with reverse transcriptase inhibitors, called highly active antiretroviral therapy (HAART), significantly enhanced the clinical outcome, reduced mortality, and morbidity, and greatly improved the life expectancy in HIV-1-infected individuals. HAART cannot eradicate the virus from HIV-infected cases. Drug-resistant, long-term use toxicity, and related high-cost limit the use of HAART in infected individuals (Simon et al., 2006). These all raise the need for novel medicinal agents (Kitazato et al., 2007).

The release of new viruses from the host cell is the final step of the virus infection cycle. Some drugs inhibit this phase through acting on the viral protein involved in such a process, which have been approved for the treatment of InfV infection (Hayden et al., 1997; Gubareva et al., 2000). The haemagglutinin of InfV A and B viruses bind to receptors with neuraminic acid (Gubareva et al., 2000). The neuraminidase (NA) enzymatic activity promotes the release of viruses by removing neuraminic acids from oligosaccharide chains of receptors (Colman, 1994). In addition to previously introduced M2 ion channel inhibitors, neuraminidase inhibitors (NAIs) are another class of anti-InfV drugs currently available (De Clercq, 2006). Most of the antiviral agents not only inhibit viral replication but also alter cellular metabolism, which creates the toxicity.

**Figure 1:**
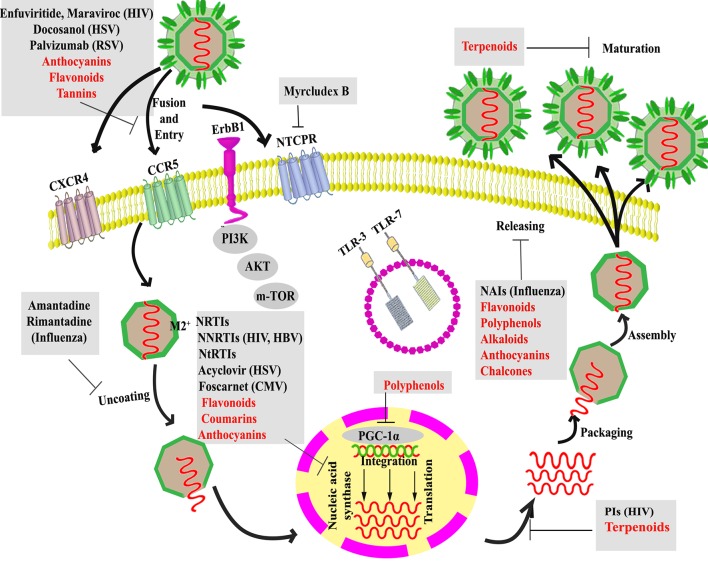
Targeting host proteins and several steps of the viral life cycle by current therapeutic and alternative natural products (in red). HIV, human immunodeficiency viruses; RSV, respiratory syncytial virus; HBV, hepatitis B virus; HSV-1/2, herpes simplex virus-1/2, NtRTIs, nucleotide reverse transcriptase inhibitors; NRTIs, nucleoside reverse transcriptase inhibitors; and NNRTIs, non-nucleoside reverse transcriptase inhibitors; NAIs, neuraminidase inhibitors; PGC-1α, peroxisome proliferator-activated receptor gamma coactivator 1-alpha.

From the antiviral drugs restrictions point of view, there is no suitable vaccine or therapeutic option for many viral infections. Conventional antiviral drugs are limited by the inadequate response, the rapid development of resistance, and adverse effects. It is well established that using even standard and the same doses of antiviral drugs may result in different inter-individual serum concentrations and clinical outcomes, the reasons for which may be multifactorial including differences in concomitant medications, underlying diseases, treatment compliance, genetic factors, and gender-related metabolism. This noticeably influences the antiviral efficacy, consequent resistant variants, and the toxicity incidence. Drug hypersensitivity reactions, which vary in clinical manifestations, severity, and frequency, are among other adverse effects of a few antiviral drugs (Tozzi, 2010; Chaponda and Pirmohamed, 2011) (**Figure 1**). Altogether, the best antiviral agents would include those more effective, having less resistance and which pose low toxicity. Therefore, the development of innovative and novel therapeutic and preventive strategies for viral infections is of particular importance. Further research is necessary to increase our knowledge about antiviral drugs adverse effects, to discover unknown mechanisms of novel therapies and to develop new antiviral agent (e.g. plant-derived) with innovative pharmacological mechanisms.

## New Targets to Combat Viral Infections: Host Proteins, Nucleic Acids, and Antibodies

In addition to previously described viral targets, new classes of antiviral drugs targeting host factors involved in virus replication, virus-cell interactions, and the immune response have been introduced.

Targeting some host proteins participating in the replication of viruses positively modulate the viral infection, and their inhibition has recently shown new insights into antiviral drugs development (Huthoff and Towers, 2008). Although not all of these proteins have been identified, recent advances in clinical studies indicated additional targets to combat viral infection. Targeting the host cell factors involved in the HIV-1 replication might be one way to overcome the resistance of HIV-1 to antiviral drugs (Opar, 2007). While drugs directed to viral proteins act against specific viral infections, those targeting host proteins have a wide range of antiviral effects (Antonelli and Turriziani, 2012). Binding the HIV envelope protein gp120 to the CD4 receptor on host cells is followed by engagement of specific chemokine receptors (CXCR4 and CCR5) as co-receptors of host cells for fusion and their antagonists considered as HIV-fusion inhibitor (Opar, 2007; Kindberg et al., 2008; Lim and Murphy, 2011). Zinc-finger nucleases, a powerful tool to edit the human genome, has also been considered as another approach making cells virtually resistant to HIV infection through knockout of CXCR4 or CCR5 receptors (Holt et al., 2010; Rahman et al., 2011; Yuan et al., 2012).

Blocking the replication through inhibiting the host tyrosine kinase (Reeves et al., 2005), and inhibiting ErbB1, a cellular growth factor, also strongly inhibited the replication of poxviruses and vaccinia virus, respectively (Yang et al., 2005).

The current studies also confirmed the role of host immune modulators toward antiviral effects, as the agonist of toll-like receptor-3 or 7 (TLR-3 or 7), showed the potential antiviral effects (Miller et al., 2008). Inhibitors of IL8 and targeting IL12, IL2, interferon-gamma (IFNγ) and tumor necrosis factor-α (TNF-α) are other possible immune-modulators used to combat some viral infections (Koumbi, 2015). It is now well known that several other therapeutic targets can improve the clinical outcome of our current standard treatment, such as programmed death 1 (PD-1), cytotoxic T-lymphocyte antigen 4 (CTLA-4), T-cell immunoglobulin and mucin domain-containing molecule 3 (TIM-3) (Khan et al., 2019), which all regulate the state of T-cell exhaustion (Hakim et al., 2014).

Therapeutic antibodies have also shown promising effects against viral infections, including RSV, HIV, avian InfV, and rabies viruses (Chen and Dimitrov, 2012).

Nowadays, ribozymes, aptamers, and RNA interference (RNAi), specifically small interfering RNAs (siRNAs) are of other nucleic acid-based antiviral therapies to target HIV, HCV, HBV, Marburg virus, Ebola virus and influenza viral genes (Zeller and Kumar, 2011). Besides, several other classes of immune modulators and antiviral agents are still under investigation (**Figure 1**).

As novel cellular targets of licensed antiviral drugs, DNA terminase, helicase, and primase have a promising future. Letermovir has shown prophylactic effects against HCV in phase 2 and 3 of a clinical trial, by influencing the synthesis of the viral terminase complex (Kropeit et al., 2017). It did not demonstrate cross-resistance with other antiviral drugs, but did demonstrate a promising efficacy against cidofovir, foscarnet, and ganciclovir-resistance viruses (Melendez and Razonable, 2015). The primary inhibitors of terminase, benzimidazole derivatives (Valiente‐Echeverría et al., 2015), were initially introduced and developed as anticancer drugs. The efficacy of these derivatives on CMV replication did not comprise the DNA synthesis inhibition on viruses but led to virions production, which had a left-end-truncated genome inside their capsid (McVoy and Nixon, 2005). The results showed that benzimidazole derivatives inhibited the replication of CMV at the final steps of viral DNA synthesis (Champier et al., 2008). The other new licensed antiviral drugs have affected the DDX3 (Asp-Glu-Ala-Asp(DEAD)-box polypeptide 3), that is a member of ATP-dependent RNA helicases. DDX3 plays various roles in RNA metabolism, such as transcription, translation, nuclear export, and stress granules assembly. Moreover, the evidence showed that DDX3 is a part of the innate immunity response against viral infections. It is interesting to note that some RNA viruses, such as HCV and HIV-1, use DDX3 to perform different replication cycles stages. Therefore, it appears that viruses have progressed to use DDX3’s boxes to coincide with threatening, so the innate immune response (Valiente‐Echeverría et al., 2015) and targeting helicase could be a novel strategy to combat viral diseases. One of the other new classes of antiviral drugs with the potential to treat HSV infections is the helicase-primase inhibitors (Kleymann et al., 2002), through affecting the non-structural protein-3 helicase (NS3h) and 5B (NS5B) RNA-dependent RNA polymerase, which are necessary for RNA replication of viruses. The helicase-primase complex is also the target of prilelivir and amenamevir with antiviral activities against HSV-1 and HSV-2 (Kleymann et al., 2002). This class of antiviral drugs also showed a potential efficacy against varicella-zoster virus (VZV) (Chono et al., 2010). Altogether, these proteins are unique targets for the discovery and development of direct-acting antivirals (Yang et al., 2017).

## The Contribution of Ethnopharmacology to the Development of Antiviral Drugs

In the past few years, some natural products and synthetic compounds had potential *in vitro* and *in vivo* antiviral activities. Among them, only a few have been approved for clinical use by western health (Vlietinck and Berghe, 1991). However, some agents have been in drug development, including both preclinical and clinical assessment, and have led to more prospects for discovering new antiviral agents with promising future. Among these antiviral substances, some are natural compounds which were isolated from medicinal plants used in complementary and traditional medicine, such as polysaccharides (Premanathan et al., 1999), polyphenols (Sokmen et al., 2005), flavonoids (Veckenstedt et al., 1978), anthocyanins (Simões et al., 2010), phenyl carboxylic acids (Kulkarni and Sanghai, 2014), terpenes (Wright et al., 1993), alkaloids (Özçelik et al., 2011), phenolic compounds (Özçelik et al., 2011), depsides (Hassan et al., 2019) and amino acids (Han et al., 1998). The number of secondary metabolites showed a unique antiviral mechanism of action and have a promising future for clinical research (Vlietinck and Berghe, 1991). There are some methods in the selection of plants for the assessment of antiviral activity, including mass screening of collected randomized plants; ethnomedical usage, available literature, and chemotaxonomical methods (Vlietinck and Berghe, 1991). Altogether, the plant kingdom is one of the best sources of new antiviral agents.

## Role of Natural Products for the Prevention and Treatment of Viral Diseases

For years, natural medicines have been used for the treatment and prophylactic of several viral infections (Koehn and Carter, 2005; Kitazato et al., 2007; Newman and Cragg, 2007). Many of the natural compounds, in particular biologically active small molecular, act as multi-target agents of high biochemical specificity and chemical diversity with lower cost and more covering mechanisms. They all help to find novel antiviral lead-compounds and lead-structures. Therefore, traditional and alternative medicinal plants offer novel promising antiviral effects.

Considerable advancement has been made in the use of several natural plant-derived products for the treatment of HIV infection. It has been previously shown that terpenoids, coumarins and flavonoids possess promising activities for the prevention and attenuation of the HIV infection. Flavonoids have been found to inhibit fusion (Li et al., 2000), integration (Lee et al., 2003), and reverse transcription (Kitamura et al., 1998). Inhibiting protease (Min et al., 1999a; Min et al., 1999b), reverse transcriptase (Rukachaisirikul et al., 2003), replication (Zhang et al., 2003), and maturation (Yu et al., 2007) are among anti-HIV mechanisms of some terpenoids. Coumarins also inhibit reverse transcriptase to show their anti-HIV effect. More recently, a tricyclic coumarin has been shown to suppress nuclear factor-kappa B (NF-кB) activation and, thereby, inhibited HIV replication *in vitro* (Kudo et al., 2013).

To date, flavonoids (Miki et al., 2007), polyphenols (Sokmen et al., 2005), alkaloids (Serkedjieva and Velcheva, 2003), anthocyanins (Krawitz et al., 2011), chalcones (Dao et al., 2011), xanthones (Dao et al., 2012), and homoisoflavonoids (Jeong et al., 2012) have also been introduced as anti-influenza agents predominantly inhibiting the NA enzyme (Wang et al., 2006).

Extensive reports have been conducted on the anti-HBV effects of natural products. A polyphenol, isochlorogenic acid (Hao et al., 2012), dehydrocheilanthifoline and some other amide alkaloids have been reported to show anti-HBV effects (Jiang et al., 2013; Zeng et al., 2013). Curcumin, as a polyphenol, down-regulated the coactivator of HBV transcription, peroxisome proliferator-activated receptor-gamma coactivator 1-α (PGC-1α), and thereby inhibited HBV gene replication and expression (Rechtman et al., 2010).

The medicinal herbs also showed noteworthy *in vitro* inhibitory activity by inhibiting protease to combat HCV (Hussein et al., 2000). Recent reports on natural products highlighted the flavonoid antiviral activity against HCV in different steps of its life cycle. Ladanein blocked the entry phase and quercetin, luteolin, and apigenin inhibited the replication phase. Honokiol, as a lignan, inhibited either entry or replication phases of HCV (Calland et al., 2012). Silymarin also inhibited HCV in different stages, including fusion, assembly (as naringenin blocked this phase) and transmission (Calland et al., 2012). It also displayed immunomodulatory and anti-inflammatory effects, contributed to its hepatoprotective effects (Morishima et al., 2010). The *Sophora* alkaloids also showed the antiviral potential to inhibit liver fibrosis, reduce the destruction of liver cells, inhibit viral replication, and promote the bile flow (Liu et al., 2003).

It is also crucial to identify novel anti-HSV lead-compounds with new mechanisms of action. Among natural entities, anthraquinone (De Logu et al., 2000), terpenoids, polyphenols, phenolics, flavonoids [e.g. houttuynoids (Houttuynoids, 2012)], proanthocyanidins (Danaher et al., 2011), geraniin (Yang et al., 2007a), hippomanin (Yang et al., 2007b), and excoecarianin (Cheng et al., 2011) have also shown promising anti-viral activities against HSV (Khan et al., 2005).

Chebulagic acid and punicalagin as tannins demonstrated an entry-inhibitor effect to HSV-1 (Chang et al., 2004). These hydrolyzable tannins inhibited viral entry phase, including attachment and penetration, thereby showing anti-RSV effects (Lin et al., 2013). New antiviral chromone glycosides (Uncinoside A) (Ma et al., 2003), biflavonoid (Genkwanol) (Huang et al., 2010), and flavone (Wang et al., 2012) protected against RSV with an unclear mechanism and resveratrol by reducing RSV-induced Inflammation through down-regulation of IFNγ levels (Zang et al., 2011). **Figure 1** also displays the mechanistic pathways which are blocked by natural products.

## Anthocyanins

Anthocyanins belong to the most conspicuous structural class of glycosylated polyphenol, which produce anthocyanidins in the underlying conditions (Castaneda-Ovando et al., 2009), and show an unlimited range of colors, associated with their specific structures (Holton and Cornish, 1995). These structures are associated with the color of plant organs such as leaves, flowers, and fruits (Pojer et al., 2013). The importance of anthocyanins is related to their multiple functions within plants. The presence of anthocyanins in petals is connected to attracting the pollinators, while the anthocyanin presence in seeds and fruits may lead to dispersal of the seed. The other importance of anthocyanins in plants is their use as feeding deterrents and protectants against UV irradiation- induced damage (Holton and Cornish, 1995).

### Chemical Structures

**Figure 2:**
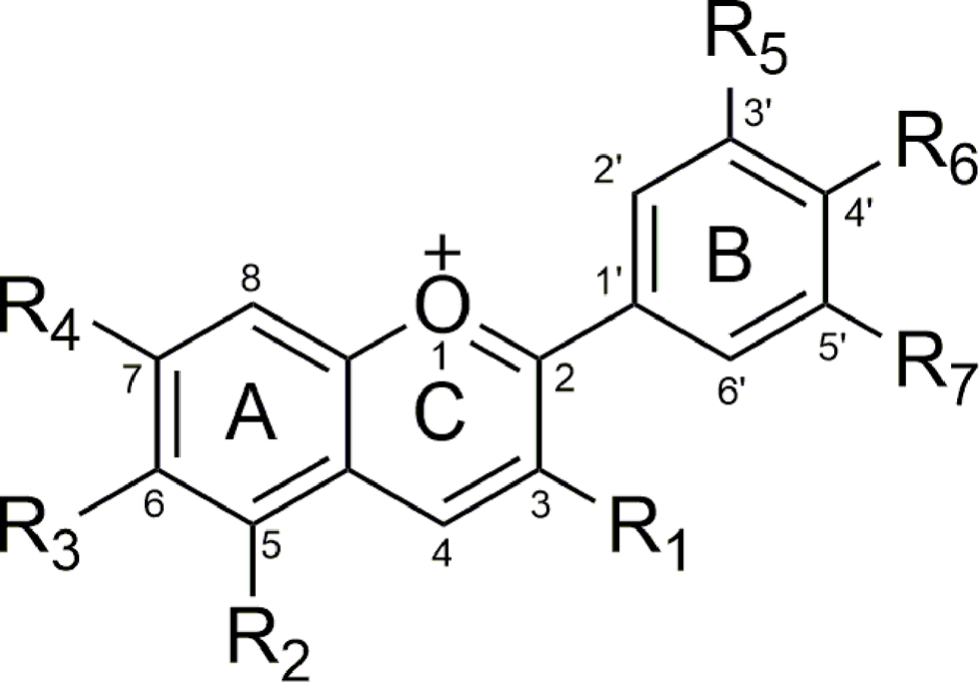
General anthocyanin’s structure.

Anthocyanidins as aglycones consisting of a benzyl ring (A) connected to a heterocyclic ring with oxygen function (C), which is also connected to a third benzyl ring (B). Part C with three carbon bridges is connected to parts A and B (**Figure 2**) (Smeriglio et al., 2016).

**Figure 3:**
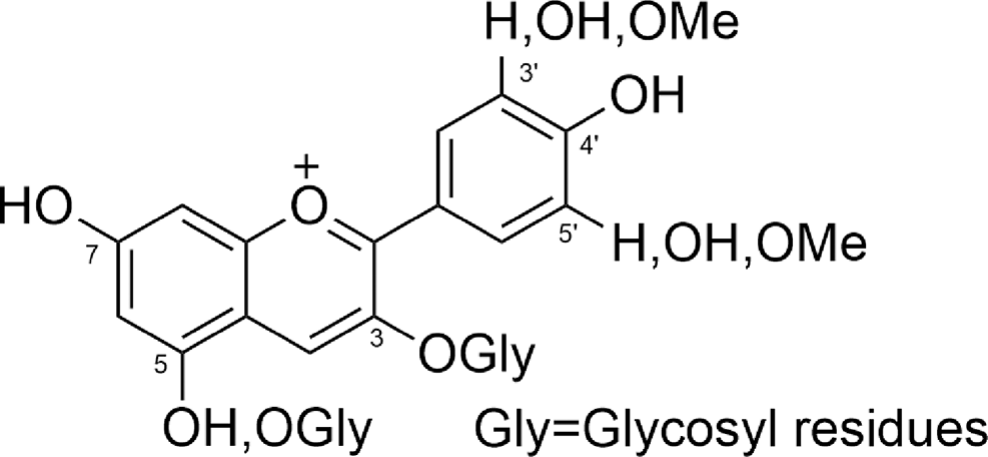
Number, position, and substituents of anthocyanins.

Glycosidic bonds are formed at 3 or 5 positions or both, and produce the monosaccharides, including glucose, galactose, rhamnose, and arabinose, as well as di-saccharides, and tri-saccharides, which could possess acylated side chains with aliphatic or cinnamic acids (**Figure 3**) (Goto, 1987).

This is the cause of the structural diversity of anthocyanins with the difference in stability and color. The fundamental differences would be the total number and position of OH groups, nature, number, and position of connected sugars, the carboxylates in aliphatic chain or aromatic forms or acylated groups linked to the sugar in the structures, and the position and place of these connections (Goto, 1987) (**Table 1**).

**Table 1:** Name, substituents, color, and the plant that anthocyanins were isolated.

Compound	R_1_	R_2_	R_3_	R_4_	R_5_	R_6_	R_7_	Color	Plant	Reference
Apigeninidin	H	OH	H	OH	H	OH	H	N.R.	*Sorghum caudatum*	(Kouda-Bonafos et al., 1994)
Arrabidin	H	H	OH	OH	H	OH	OMe	N.R.	*Arrabidaea chica*	(Wouters et al., 2005)
Aurantinidin	OH	OH	OH	OH	H	OH	H	N.R.	*Alstroemeria*	(Tatsuzawa et al., 2003)
Capensinin	*O*-rhamnose	OMe	H	OH	OMe	OH	OMe	N.R.	*Plumbago* and *Ceratostigma*	(Harborne, 1967)
Carajurin	H	H	OH	OH	H	Ome	OMe	N.R.	*Arrabidaea chica*	(Wouters et al., 2005)
cyanidin-3-*O*-glucoside	*O*-glucoside	OH	H	OH	OH	OH	H	Orange-red	Purple rice and pomegranate	Diaz-Mula et al., 2019; Wongwichai et al., 2019)
Cyanidin-coumaroyl-hexoside	coumaroyl-hexoside	OH	H	OH	OH	OH	H	N.R.	Grape	(Colombo et al., 2019)
cyanidin 3-rutinoside	rutinoside	OH	H	OH	OH	OH	H	N.R.	*Prunus spinosa*	(Leichtweis et al., 2019)
Delphinidin-3-*O*-glucoside	*O*-glucoside	OH	H	OH	OH	OH	OH	Blue-red	Grape	(Colombo et al., 2019)
Delphinidin-coumaroyl-hexoside	coumaroyl-hexoside	OH	H	OH	OH	OH	OH	N.R.	Grape	(Colombo et al., 2019)
Delphinidin-caffeoyl-hexoside	caffeoyl-hexoside	OH	H	OH	OH	OH	OH	N.R.	Grape	(Colombo et al., 2019)
Delphinidin-acetyl-hexoside	acetyl-hexoside	OH	H	OH	OH	OH	OH	N.R.	Grape	(Colombo et al., 2019)
Europinidin	OH	OMe	H	OH	OMe	OH	OH	Blue-red	*Plumbago europea*	(Harborne, 1967)
Hirsutidin	OH	OH	H	OMe	OMe	OH	OMe	Blue-red	*Catharanthus roseus* and *Primula sp*.	(Iwashina, 2000)
3′-Hydroxyarrabidin	H	H	OH	OH	OH	OH	OMe	N.R.	*Arrabidaea chica*	(Wouters et al., 2005)
6-Hydroxycyanidin	OH	OH	OH	OH	OH	OH	OH	Red	*Alstroemeria*	(Saito et al., 1985)
6-Hydroxydelphinidin	OH	OH	OH	OH	OH	OH	OH	Blue-red	*Alstroemeria*	(Nørbaek et al., 1996)
6-Hydroxyplargonidin	OH	OH	OH	OH	H	OH	H	N.R.	*Alstroemeria*	(Tatsuzawa et al., 2003)
Malvidin-3-*O*-glucoside	*O*-glucoside	OH	H	OH	OMe	OH	OMe	Blue-red	Grape	(Colombo et al., 2019)
Malvidin-caffeoyl-hexoside	caffeoyl-hexoside	OH	H	OH	OMe	OH	OMe	N.R.	Grape	(Colombo et al., 2019)
Malvidin-cumaroyl-hexoside	cumaroyl-hexoside	OH	H	OH	OMe	OH	OMe	N.R.	Grape	(Colombo et al., 2019)
Malvidin-acetyl-hexoside	acetyl-hexoside	OH	H	OH	OMe	OH	OMe	N.R.	Grape	(Colombo et al., 2019)
5-Methylcyanidin	OH	OMe	H	OH	OH	OH	H	Orange-red	cashew apple	(de Britoa et al., 2007)
Pelargonidin 3-*O*-rutinoside	*O*-rutinoside	OH	H	OH	H	OH	H	N.R.	Gentian	(Diretto et al., 2019)
Pelargonidin 3-*O*-(6-*p*-coumaroyl) glucoside	*O*-(6-*p*-coumaroyl)glucoside	OH	H	OH	H	OH	H	N.R.	Gentian	(Diretto et al., 2019)
Pelargonidin 3-*O*-glucoside	*O*-glucoside	OH	H	OH	H	OH	H	N.R.	Gentian	(Diretto et al., 2019)
Pelargonidin 3-*O*-rutinoside-5-*O*-β-D-glucoside	*O*-rutinoside	*O*-β-D-glucoside	H	OH	H	OH	H	N.R.	Gentian	(Diretto et al., 2019)
Pelargonidin 3,5-*O*-diglucoside	*O*-diglucoside	*O*-diglucoside	H	OH	H	OH	H	N.R.	Gentian	(Diretto et al., 2019)
peonidin-3-*O*-glucoside	*O*-glucoside	OH	H	OH	OMe	OH	H	N.R.	Purple rice and Grape	(Colombo et al., 2019; Wongwichai et al., 2019)
Peonidin-coumaroyl-hexoside	coumaroyl-hexoside	OH	H	OH	OMe	OH	H	N.R.	Grape	(Colombo et al., 2019)
Peonidin-caffeoyl-hexoside	caffeoyl-hexoside	OH	H	OH	OMe	OH	H	N.R.	Grape	(Colombo et al., 2019)
Peonidin-acetyl-hexoside	acetyl-hexoside	OH	H	OH	OMe	OH	H	N.R.	Grape	(Colombo et al., 2019)
peonidin 3-rutinoside	rutinoside	OH	H	OH	OMe	OH	H	N.R.	*Prunus spinosa*	(Leichtweis et al., 2019)
Peonidin -3-(6″-caffeoyl-6?- feruolylsophoroside)-5-glucose	-(6″-caffeoyl-6?- feruolylsophoroside)	glucose	H	OH	OMe	OH	H	N.R.	Sweet Potato	(Vishnu et al., 2019)
Petunidin-3-*O*-glucoside	*O*-glucoside	OH	H	OH	OMe	OH	OH	Blue-red	Grape	(Colombo et al., 2019)
Petunidin-coumaroyl-hexoside	coumaroyl-hexoside	OH	H	OH	OMe	OH	OH	N.R.	Grape	(Colombo et al., 2019)
Petunidin-caffeoyl-hexoside	caffeoyl-hexoside	OH	H	OH	OMe	OH	OH	N.R.	Grape	(Colombo et al., 2019)
Petunidin-acetyl-hexoside	acetyl-hexoside	OH	H	OH	OMe	OH	OH	N.R.	Grape	(Colombo et al., 2019)
Pulchellidin	OH	OMe	H	OH	OH	OH	OH	Blue-red	*Plumbago pulchella*	(Iwashina, 2000)
Riccionidin A	OH	H	OH	OH	H	OH	H	N.R.	*Ricciocarpos natans*	(Iwashina, 2000)
Rosinidin	OH	OH	H	OMe	OMe	OH	H	Red	*Primula rosea*	(Iwashina, 2000)
Tricetinidin	H	OH	H	OH	OH	OH	OH	Red	black tea	(Iwashina, 2000)

**Figure 4:**
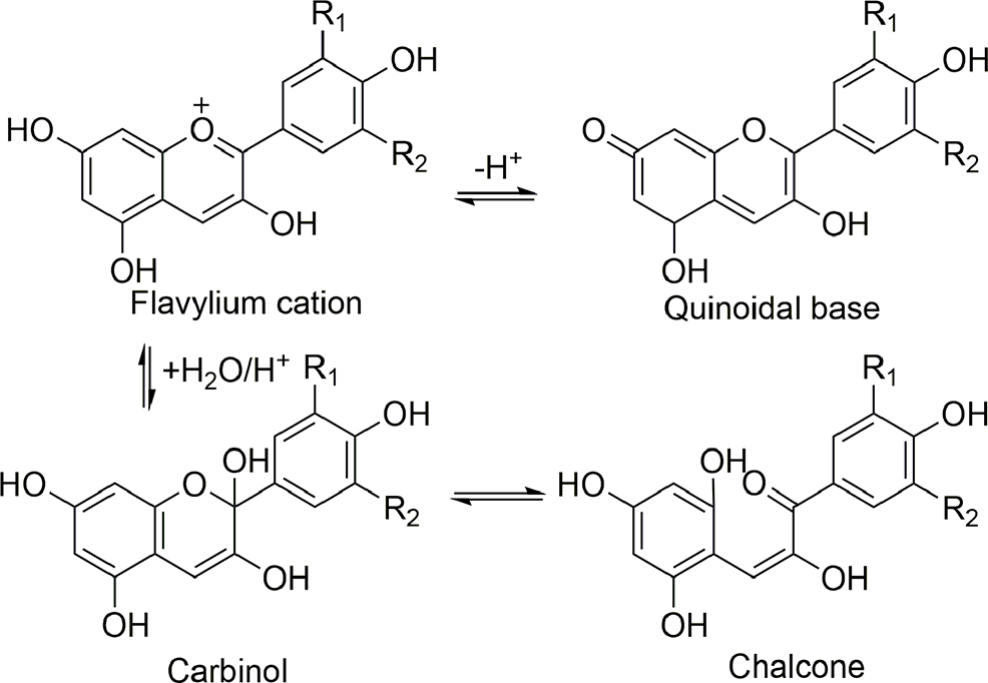
The substitution pattern of flavylium cation forming the naturally-occurring anthocyanidins which are known today.

The sugar residues of anthocyanin may be acylated by caffeic, sinapic, *p*-coumaric, and ferulic acids, and occasionally by acetic acid, malonic acid or *p*-hydroxybenzoic acid. The position of the C-3 in sugar is sometimes substituted by acyl (Brouillard, 1982; Goto, 1987). The most crucial characteristic of anthocyanins is their color changes against pH variation (Goto, 1987). The acidic pH of anthocyanins causes the following principal equilibrium types, including the quinonoidal base, which captures proton and produces the flavylium cation with a resonating system, picking up H_2_O and leading the reaction for producing the anhydrobase or carbinol, and the chalcone types (Rein, 2005; Swaminathan et al., 2014) (**Figure 4**). The stability of anthocyanins depends on major parameters like oxygen, chemical structure, pH, light, solvents, concentration, processing and storage temperature, the presence of proteins, enzymes, metallic ions, flavonoids, and presentment of ascorbic acid preserve from sugars and oxidation (Rein, 2005).

### Biological Activities and Health Benefits of Anthocyanins

Anthocyanins are indeed among the critical supplements that improve human health (Yeung et al., 2018) and play a functional antioxidant activity through free-radical scavenging, decreasing ROS and lipid peroxidation (Wang et al., 1999; Lila, 2004). Anthocyanins supply a cardioprotective effect *via* increasing the postischemic coronary flow, inhibiting of the cholesteryl ester transfer and consequently regulating the serum level of LDL and HDL (Qin et al., 2009), as well as the occurrence and period of reperfusion arrhythmias (Qin et al., 2009; Pojer et al., 2013). Anthocyanin-rich formula inhibited H_2_O_2_- and TNF-α-induced vascular endothelial growth factor release (Bagchi et al., 2004), and activated both caspase-dependent and independent pathways (Reddivari et al., 2007), thereby exerting its anti-angiogenic and anticarcinogenic activities.

Anthocyanins also improved the visual function through the inhibition of transitory myopia, improving the blood flow of retin, enhancing dark adaptation, and decreasing eye fatigue in glaucoma disease (Tsuda, 2012). From the neuroprotective point of view, anthocyanins improved the brain function through enhancement of the memory and motor performances (Tsuda, 2012), enabling the cunctation of the onset of age-related neurodegenerative dysfunction (Pojer et al., 2013). Anthocyanins also displayed anti-obesity and anti-diabetic activity (increasing the insulin secretion) (Jayaprakasam et al., 2005), anti‐inflammatory activity (through modification of inflammatory cytokines and mediators including IL-1, IL-6, IL-10, TNF-a, NF-kB, inducible nitric oxide synthase and COX-2)(Bowen-Forbes et al., 2010; Sodagari et al., 2015) and bacteriostatic activity (Cisowska et al., 2011). Previously, anthocyanins were shown to have pharmacological and nutraceutical activities and also played a key role in human nutrition that improves both physical and mental health.

### Anthocyanin Enriched Plant and Fruits With Antiviral Effect

The antiviral activity of anthocyanins is considered to be one of their pharmacological properties, which indicates their importance in health and hygiene. Furthermore, the antiviral effects of anthocyanin-enriched plants and fruits have so far been reported (**Table 2**).

**Table 2:** The antiviral activity of anthocyanins-enriched plants and purified anthocyanins with their pharmacological activity.

Antiviral activity against	Active compounds	Plant material	Mechanism of action	Ref
Anthocyanins-enriched plants				
InfV A and B	3-*O*-α-L-rhamnopyranosyl-β-D-glucopyranosyl-cyanidin, 3-*O*-β-D-glucopyranosyl-cyanidin, 3-*O*-α-L-rhamnopyranosyl-β-D-glucopyranosyl-delphinidin, and 3-*O*-β-D-glucopyranosyl-delphinidin	*Ribes nigrum*	Inhibited virus adsorption to cells and also virus release from infected cells	(Knox et al., 2001)
InfV A and B	Pelanin, pelargonidin, pelargonidin 3-*p*-coumaroylglucose- 5-glucose, pelargonidin 3-*p*-coumaroylglucose- 5-malonylglucose	*Solanum tuberosum*	The structure of the anthocyanin pigment, their additive or synergistic effects and other coexisting constituents in the tuber.	(Hayashi et al., 2003)
InfV	Cyanidin-3-sambubiocide	*Sambucus nigra *	Binding to 430-cavity near the neuraminidase residues to regulate neuraminidase resistance.	(Swaminathan et al., 2013)
HSV-1	Delphinidin-3-rutinoside	*Solanum melongena*	Inhibite the HSV-1 replication, reduce viral proteins expression, and reduce NOX4 expression	(Di Sotto et al., 2018)
HSV-1	Total anthocyanins	*Fragaria x ananassa *	Increase the anthocyanins content and decrease the detected anti-HSV-1 activity	(Simões et al., 2012)
RV	Anthocyanin-related substance	*Vigna angularis *	Effect on the early phase of the infection cycle	(Kawai and Fujita, 2007)
InfV A and CV-B1	Total anthocyanins	*Fragaria vesca, Rubus idaeus, Vaccinium myrtillis, and V. vitis-idaea *	Inhibit the replication of viruses	(Nikolaeva-Glomb et al., 2014)
InfV A and B	Cyanidin-3-rutinoside	*Morus alba*	Inhibite the attachment phase or internalization and intervene with the ROS-mediated cell damage caused by InfV Infection	(Kim and Chung, 2018)
Adenovirus 36	Kuromanin chloride and total extract	*Morus alba*	Inhibits viral replication	(Na et al., 2014)
BT2, T4 and simian rotavirus SA-11	Total anthocyanins	*Vaccinium macrocarpon*	Inhibited the viral adsorption of phage T4 and the replication of rotavirus	(Lipson et al., 2007)
HAV, FCV-F9 and MNV-1	Total anthocyanins	*Vitis vinifera*	Inhibite the viral adsorption and have less effect on replication	(Joshi et al., 2015)
InfV A, InfV B and avian InfV.	cyanidin-3-arabinoside and cyanidin-3-galactoside	*Theobroma cacao*	Increase the NK cell activity, enhance the immune system responses and prepare more potent protection	(Niemenak et al., 2006)
InfV A	Total anthocyanins	*Lycium barbarum *	Attenuate the inflammatory cytokines in the lungs and raise T cells mediated function by producing IL-2	(Ren et al., 2012)
InfV subtypes A/Vama/5/003 and A/Vama/3/003.	Total anthocyanins	*Aronia melanocarpa*	Inhibit the reproduction of InfV in its initial phase	(Valcheva-Kuzmanova et al., 2003)
Adenovirus and HIV-1 IIIB	Total anthocyanins	*Punica granatum*	Bind irreversibly or strongly to the binding site of CD4 on gp120	(Neurath et al., 2004)
HSV-1, HSV-2, and ACV^res^	Total anthocyanins	*Lamiaceae family*	Inhibit the virus adsorption stage and have no effect on the replication stage	(Nolkemper et al., 2006)
**Purified antiviral anthocyanin**				
HCV	Delphinidin		Inhibit the attachment stage	(Calland et al., 2015)
InfV	Cyanidin-3-sambubiocide		Regulate neuraminidase resistance	(Kannan and Kolandaivel, 2018)
WNV, DENV, and ZIKV	Delphinidin, and cyanidin		Inhibit the attachment, adsorption and entrance stages and a have direct activity on virus particles	(Vázquez-Calvo et al., 2017)
InfV neuraminidase	Pelargonidin, cyanidin and delphinidin		Depend on OH substituent each compound with more number of OH substituent, bond strongly and have a more inhibitory activity	(Swaminathan et al., 2014)

#### *Ribes nigrum* L.

Some functional anthocyanins such 3-*O*-rutinosides and 3-*O*-glucosides of malvidin, pelargonidin, peonidin, petunidin, delphinidin, cyanidin, and cyanidin 3-*O*-arabinoside have been isolated from the extract of blackcurrant berries (*Ribes nigrum* L.) (Slimestad and Solheim, 2002). Knox et al. studied the effect of *R. nigrum* extract against the InfV A and B and showed four anthocyanins-containing fractions with potent antiviral activity. According to the results, one of the fractions contained 3-*O*-α-L-rhamnopyranosyl-β-D-glucopyranosyl-cyanidin and 3-*O*-β-D-glucopyranosyl-cyanidin anthocyanins, and the second contained 3-*O*-α-L-rhamnopyranosyl-β-D-glucopyranosyl-delphinidin and 3-*O*-β-D-glucopyranosyl-delphinidin. Both fractions displayed strong antiviral activity against the InfV A and B. The second fraction anthocyanins prevented viral attachment and entry to cells, as well as their release from the infected cells (Knox et al., 2001). *R. nigrum* extracts also inhibited InfV adsorption on the cell surface (Ikuta et al., 2013), interference with virus internalization (Ehrhardt et al., 2013) and completely suppressed the growth of InfV A *in vitro* (Knox et al., 2003). As Ikuta et al. reported, a low concentration of blackcurrant inhibited the replication while a high one inhibited adsorption of InfV A and B onto the cell surface (Ikuta et al., 2012). Altogether, the blackcurrant extracts displayed auspicious anti-InfV effects through different mechanisms. This extract might be a hopeful source for the drug discovery and development of new antiviral agents to struggle against InfV infections.

#### *Solanum* spp.

As red potatoes, *Solanum stenotomum* and *S. tuberosum* were surveyed potential sources for natural red colorants, especially anthocyanins and contained pelargonidin-3-rutinoside-5-glucoside acylated with *p*-coumaric acid as major anthocyanins (Rodriguez‐Saona et al., 1998). Only the anthocyanins of red-fleshed potato were observed to possess antiviral effects till now (Zhao et al., 2009). Hayashi et al. investigated the activity of red-fleshed potato anthocyanins against the InfV A and B viruses. According to the results, the purified *Solanum* sp. anthocyanin, pelanin, pelargonidin, pelargonidin 3-*p*-coumaroylglucose-5-malonylglucose, and pelargonidin 3-*p*-coumaroylglucose-5-glucose were effective against the InfV B virus. They also reported that the inhibitory impact of anthocyanins against InfV A and B viruses arose from their molecular structure, synergistic effects, and coexisting components of tuber (Hayashi et al., 2003; Zhao et al., 2009). Valadares et al. investigated the antiviral effect of *S. paniculatum* against murine encephalomyocarditis virus (EMCv), human herpes virus type 1 (HHV-1), and vaccinia virus strains Western Reserve (VACV-WR). According to the results, the extract of *S. paniculatum* inhibited the replication of HHV-1, but exhibited no effect on VACV-WR and EMCv (Valadares et al., 2009). Javid et al. also studied the anti HCV activity of chloroform extract of *S. nigrum* seeds (SNS). As reported by the authors, the chloroform extract of SNS reduced the expression or function of HCV NS3 protease in a dose-dependent manner. Furthermore, NS3 may have extra effects involved in interference with host cell functions, like inhibition of protein kinase A-mediated signal transduction or cell transformation. Besides, the antiviral activity of SNS is a target for therapeutic development of new antiviral compounds for acute and chronic HCV, where NS3- mediated processing of the polyprotein is essential for HCV RNA replication and maturation (Javed et al., 2011). Therefore, the antiviral effects of *Solanum* sp. anthocyanins are believed to belong to the synergistic or additive effect of them with other coexisting pigments, which inhibit the replication and decrease the expression of protease for RNA replication and maturation. Therefore, this species is a conspicuous source of antiviral agents and could become a structure template for the development of new antiviral therapy.

#### *Fragaria × ananassa* (Duchesne ex Weston) Duchesne ex Rozier - Strawberries

An analysis of the anthocyanins of strawberry varieties reported by Garcia‐Viguera et al. introduced cyanidin 3-glucoside, pelargonidin 3-glucoside, pelargonidin 3-rutinoside in fresh and frozen fruit (Garcia‐Viguera et al., 1998). In the 1970s, researchers reported the antiviral activity of strawberry fruit juice against coxsackievirus B5, poliovirus type 1 and echovirus 7 (Konowalchuk and Speirs, 1976). Carvalho et al. studied five strawberry cultivars and quantified their total anthocyanins content (TA). The extracts were evaluated for in vitro anti-HSV activity. They showed that aromas and camarosa cultivars had the highest TA, but camino real cultivar presented the lowest TA. The two first cultivars also showed the most activity against the herpes virus, while the camino real cultivar had the lowest activity. Finally, the authors suggested a relationship between the amount of anthocyanins and the detected anti-HSV for the strawberry’s cultivars (Simões et al., 2012). In another research study by Willig et al., the anti-HSV-1 activity of strawberry’s anthocyanins were examined. They revealed that the concentrations equal to or greater than 20µg/ml of strawberry’s anthocyanins completely inhibited HSV-1 (Willig, 2013). In summary, strawberry anthocyanins are effective prophylactic agents to treat or tackle HSV-1.

#### *Vigna angularis* (Willd.) Ohwi & H.Ohashi

The isolation and structural elucidation of anthocyanin from the *Vigna angularis* (red bean) have shown cyanin as a major anthocyanin of this species of edible legumes (Yoshida et al., 1996). Kawai et al. studied different extracts of *V. angularis*, which among exudate fluids and aqueous extract, respectively, indicated potent and little antiviral activities against rabies virus (RV). According to the findings, the anthocyanins fractions were involved in the antiviral activity of *V. angularis*, which were affected by maceration conditions, and the anthocyanin-deficient white bean did not show antiviral effects into the exudate fluids. Moreover, the antiviral activity was still observed after heating for 10 min. Further studies on the antiviral activity of the extract affect preliminary adsorption phase and the preliminary phase of viral replication. The antiviral activity of *V. angularis* was not only restricted to RV, but the HSV and InfV were affected as well (Kawai and Fujita, 2007). This implicated the possible application of Adzuki bean (small red bean) exudate fluids for post-exposure treatment of rabid dog-bite injuries accompanied by vaccination.

#### Wilberries

Cyanidin, peonidin, pelargonidin, delphinidin, petunidin and malvidin glycosides as a wide variety of anthocyanins were isolated and quantified in the 24 wild and cultivated berry fruit species using high-performance liquid chromatography-diode array detector (HPLC-DAD) (Veberic et al., 2015). In spite of noticeable antimicrobial effects of wild berries, their antiviral capacity has not been well assessed (Konowalchuk and Speirs, 1976). In their study, Glomb et al. examined the methanol extract of wild berries (raspberry and strawberry of the Rosaceae, and lingonberry and bilberry of the Ericaceae family) against the representatives of Orthomyxo-, Paramyxo- and Picorna-viridae. The antiviral effects of methanolic extract, anthocyanin and non-anthocyanin fractions were evaluated on Picornaviridae [e.g., coxsackievirus B1 (CV-B1) and poliovirus type 1 (PV-1)], Paramyxoviridae (e.g. human respiratory syncytial virus A2 (HRSV-A2)), and Orthomyxoviridae such as InfV A. According to the results, the methanolic extracts of all types of berries inhibited the replication of InfV A and CV-B1. The total extract of strawberry and bilberry inhibited the highest degree of CV-B1 replication. Lingonberry, strawberry, and bilberry extracts also inhibited the InfV A virus. All of the anthocyanins fractions of wild berries had a potent effect and inhibited the replication of InfV A; however, they did not show any significant activity against the other tested viruses (Nikolaeva-Glomb et al., 2014). A straight relationship was perceived between the antiviral activity of berry extracts and total polyphenol content (Sekizawa et al., 2013). Searches demonstrated that components of berry fruits might inhibit replication of the virus even directly and indirectly, first by blocking surface gps of InfV and second by stimulating the organism immune system (Gramza-Michałowska et al., 2017). Considering the attained results, wildberry is an auspicious source of anthocyanins to combat viral infections.

#### *Rubus coreanus* Miq.

The outcomes of HPLC-PDA-MS/MS analysis of the anthocyanins extracted from *Rubus coreanus* (Bokbunja, black raspberry) exhibited the presence of some anthocyanin structures, which were tentatively identified as cyanidin 3-*O*-xylosylrutinoside, 3-*O*-sambubioside, cyanidin 3-*O*-rutinoside, delphinidin 3-*O*-rutinoside, delphinidin 3-*O*-glucuronide and pelargonidin 3-*O*-rutinoside (Ku and Mun, 2008). In their study on the black raspberry (RCS) seed enriched with a high amount of polyphenolic compounds, Lee et al. reported the antiviral activity of RCS fraction (RCS-F1) and the total extract against food-borne viral feline calicivirus-F9 (FCV-F9), murine norovirus-1 (MNV-1), and surrogates (FBVS). The highest antiviral activity was reported when both the extract and fractions were added together to infected cells with FCV-F9 or MNV-1, which resulted in a complete inhibition. The results showed that the RCS-F1 disrupted viral capsids and their expansion, and impaired the attachment to the surface protein of host cells. Moreover, one anthocyanin called cyanidin-3-glucoside (C3G) was isolated from RCS-F1 that showed inhibitory activity against the viruses. The mechanism of action of C3G is through binding to RNA polymerase of MNV-1 and expansion of viral capsids (Lee et al., 2016a). In BALB/c mice, black raspberry also reduced the viral titers of Inflv A and B in the lungs and improved the survival rate (Lee et al., 2016b), which may be related to the anthocyanins as sources of active ingredients target viruses. As Oh reported, RCS inhibited noroviruses and caliciviruses infection, probably through inhibiting their entry or attachment to the cellular receptor (Oh et al., 2012). In another study, RCS was found to exhibit antiviral activities against InfV A and B. The findings showed that RCS binds to hemagglutinin protein, significantly inhibits hemagglutination and disrupts viral particles (Lee et al., 2016b). In general, such research provided *R. coreanus* as a prospective plant and its cyanidin derivatives with potential use in the prevention and treatment of viral disease.

#### *Morus alba* L.

Cyanidin hexoside, cyanidin rhamnosylhexoside, cyanidin galloylhexoside, petunidin rhamnosylhexoside, pelargonidin hexoside, delphinidin hexoside, and delphinidin acetylhexoside as a part of anthocyanins, were reported in the extracts of mulberry (known as *Morus alba* (MA)) fruits with different distributions and contents (Natić et al., 2015). MA belongs to the Moraceae family, with antiviral activity to combat hepatitis B and C viruses (Jacob et al., 2007). Kim et al. studied the antiviral activities of MA and its juice (MAJ) and seed (MAS). MAJ fixed the glutathion (GSH) levels in the infected cells and showed ferric ion-reducing and DPPH radical scavenging activities. In the phenolic compound of MAJ, cyanidin-3-rutinoside had the highest amount and indicated a weak inhibition against InfV B in pre-treatment stage. The suggested mechanism of action of this extract against InfV would be a direct inhibition at the phase of attachment or internalization and intervention with the ROS-mediated cell damage caused by InfV infection. Finally, the results suggested that MAJ was more potential to be considered as a lead compound with antiviral activity against pre- and co-treatment but not post-treatment stage of InfV with no cytotoxicity against MDCK cells (Kim and Chung, 2018). In other research, MAS and MAJ also showed antiviral effects against foodborne viral surrogates (Lee et al., 2014). Nam et al. studied the anti-inflammatory properties of mulberry extract (ME) against adenovirus 36 (Ad36) in mice. The mixture of ME compound (1-deoxynojirimycin, kuromanin chloride, and resveratrol) and ME inhibited the virus replication by 50% and 70%, in contrast with the control group. Moreover, the ME reduced the pro-inflammatory cytokines, like monocyte chemoattractant protein‐1 (MCP-1) and TNF-α, and also decreased the concentration of macrophages and infiltrating immune cells in the fat layers of epiderm. Therefore, it can be concluded that the dietary ME might lead to prevention or treatment of obesity and inflammation caused by Ad36 (Na et al., 2014). Therefore, the authors asserted that MAJ might be useful in the prevention of foodborne viral infection, treatment of obesity and inflammation caused by Ad36 and may have a prosperous future in the prevention and treatment of viral disease.

#### *Vaccinium macrocarpon* Aiton

Several studies revealed the antiviral properties of cranberry (*Vaccinium macrocarpon*) (Nijveldt et al., 2001; Côté et al., 2010). Cyanidin-3-galactoside, cyanidin-3-glucoside, cyanidin-3-arabinoside, peonidin-3-galactoside, peonidin-3-glucoside, and peonidin-3-arabinoside were reported as the anthocyanin content of four cultivars of cranberry by P. Viskelis et al. (2009). In their study, Lipson et al. assessed the antiviral effects of cranberry juice (CJ) on unconnected viral species. The treatment of bacteriophage T2 (BT2) with CJ showed no detectable virus infection. Like BT2, almost identical data were reported for the bacteriophage T4 (BT4). The inactivation of BT4 by CJ was fast and dose dependent. Further studies determined the antiviral effect of CJ against a simian rotavirus SA-11. CJ is closely connected to the adsorption phase of the replication in virus. In addition, inhibition of hemagglutinin occurred at a 20% concentration of CJ that was treated with simian rotavirus SA-11 (Lipson et al., 2007). In another study, CJ inhibited InfV A and B adhesion to cells, and subsequent infectivity (Weiss et al., 2005). As an antiviral agent, CJ suggested being considered as an effective viral system to that of a mammalian enteric virus. The findings indicate that the inhibitory effect of CJ on InfV adhesion and infectivity may have therapeutic potential and may be a suitable natural extract for viral diseases.

#### *Vitis vinifera* L.

Delphinidin, cyanidin, petunidin, peonidin, malvidin, and their glycosides were introduced as major anthocyanin composition of fruit of *Vitis vinifera* (grape) (Cortell et al., 2007). In a research study on grape seed extract (GSE), Joshi et al. examined its antiviral effects against three viruses: feline calicivirus (FCV-F9), murine norovirus (MNV-1), and hepatitis A virus (HAV). They also studied the activity of GSE in 2% milk, apple juice (AJ), gastric juice (GJ), and water conditions at 37 °C. The antiviral activity of GSE was dose-dependent and enhanced with time. The GSE possessed some mild activities at the viral adsorption stage of viruses with minor activity on the replication stage. GSE also showed positive effects on foodborne viral diseases (Joshi et al., 2015). Previously, GSE was evaluated on the adsorption and replication phases of MNV-1, FCV-F9, and HAV prior to a viral infection was evaluated for GSE, which resulted in the decrement of viruses titer (Su and D’Souza, 2011). Similarly, GSE meaningfully down-regulated the expression of CCR2,3,5 as coreceptors of HIV entry, which interfered with the binding of the viruses and their entry into the cells (Nair et al., 2002a). In the other study, GSE indicated antiviral effects by producing Th1-derived cytokine interferon- γ (IFN-γ) through peripheral mononuclear cells, proposing that the effective immunostimulatory activity of GSE might be mediated through IFN-γ induction (Nair et al., 2002b). Therefore, considering the cost-effectiveness and broad-spectrum effects of GSE showed potential for use in the viral infections and also food quality and safety.

#### *Theobroma cacao* L.

Cyanidin-3-arabinoside and cyanidin-3-galactoside were found as two major anthocyanins in cocoa seeds (CS), which contained 0.02–0.4% of defatted CS powder (Niemenak et al., 2006). Kamei et al. investigated the antiviral activity of CS and showed which it was dose-dependently effective against, InfV A, InfV B, and avian InfV. The extract’s mechanism of action was the inhibition of the adsorption phase of viruses. In a mice model, CS significantly increased the survival after exposure to an intra-nasal lethal dose of InfV. On the other hand and to examine the antiviral properties of CS in the human model, the natural killer (NK) cell activity and the titers of neutralizing antibody against InfV A were evaluated. The results suggested that the NK cell activity was increased; though the improvement was more considerable in the treated group. The titers of neutralizing antibody were considerably enhanced in treated and control groups; however, the enhancements were not significantly different between the groups. Finally, CS and its derivatives improved the immune system responses and procured more potent protection against InfV disease (Kamei et al., 2016). In another study, the anti-HIV activity of cacao husk was evaluated. The finding showed cacao husk extract inhibited the HIV-1 cytopathic effect against MT-2 and MT-4 (HTLV-1 transformed T-cell lines), syncytium formation between HIV-infected and uninfected MOLT-4 cell line (Unten et al., 1991). Moreover, other research has shown the anti-inflammatory effects of cocoa produces (Di Giuseppe et al., 2008; Selmi et al., 2008). Altogether, according to the results, T. cacao is a promising plant containing anthocyanins to tackle viral infections.

#### *Lycium barbarum* L.

The anthocyanin content of *Lycium barbarum* (wolfberry) were reported by Zheng et al., including petunidin-3-*O*-galactoside-5-*O*-glucoside, petunidin-3-*O*-glucoside-5-*O*-glucoside, delphinidin-3-*O*-rutinoside(*cis*-*p*-coumaroyl)-5-*O*-glucoside, delphinidin-3-*O*-rutinoside (*trans*-*p*-coumaroyl)-5-*O*-glucoside, petunidin-3-*O*-rutinoside (caffeoyl)-5-*O*-glucoside, petunidin-3-*O*-rutinoside (*cis*-*p*-coumaroyl)-5-*O*-glucoside, petunidin-3-*O*-rutinoside (*trans*-*p*-coumaroyl acid)-5-*O*-glucoside, petunidin-3-*O*-glucoside (malonyl)-5-*O*-glucoside, petunidin-3-*O*-rutinoside (feruloyl)-5-*O*-glucoside, and malvidin-3-*O*-rutinoside (*cis*-*p*-coumaroyl)-5-*O*-glucoside (Zheng et al., 2011). Studies have shown the several health-promoting properties of wolfberry (Potterat, 2010). In their trial, Ren et al. studied the effect of *L. barbarum* on the improvement of mice immune system response. According to the results, lacto-wolfberry (LWB) supplementation reduced the pathology and symptoms of InfV infection through attenuation of inflammatory cytokines, such as TNFα and IL-6, in the mice that were nourished with LWB for four weeks and thereupon treated with InfV A while maintaining the same diet. However, it raised IL-2 through increasing the systemic lymphocyte T (Ren et al., 2012). In this regard, other reports have also consisted of the modulatory effects of wolfberry on the immune system (Kim et al., 2000; Li et al., 2011). The immunomodulating properties of wolfberry may increase the host’s defense against viral infection; though, the scientific mark is lacking.

#### *Aronia melanocarpa* (Michx.) Elliott

Cyanidin 3-*O*-galactoside, cyanidin 3-*O*-glucoside, cyanidin 3-*O*-arabinoside, and cyanidin 3-*O*- xyloside were of the anthocyanins from fruits of chokeberry (*Aronia melanocarpa*) and were revealed by Oszmiansk and Sapis (1988). The most-observed anthocyanins in *A. melanocarpa* fruit juice (AMFJ) were cyanidins, which were mainly in the form of aglycone (Valcheva-Kuzmanova et al., 2003). Valcheva-Kuzmanova et al. reported that AMFJ, rich in anthocyanins, decreased the activity of hemagglutination of two InfV subtypes. The OH group in location 3 was shown to be responsible for the pharmacological antiviral mechanism of *A. melanocarpa* anthocyanins to the highest inhibition level of HSV-1 replication (Wleklik et al., 1988; Bae et al., 2000). According to the results, the complex formation between the virion and anthocyanins influenced the adsorption stage of InfV infection (Valcheva-Kuzmanova et al., 2003). In another study, the anti-infV activity of chokeberry was evaluated. This plant showed cross-reactivity against InfV through anti-hemagglutinin activity by masking the hemagglutinin head in a non-specific circumstance. It is also inhibiting the viral proteins, which are impressing the maturation by blocking cellular signaling pathway (Park et al., 2013). So, in viral infections, promising effects might be expected from AMFJ through stimulation of the immune system.

#### *Punica granatum* L.

Pomegranate (*Punica granatum*) is a rich source of anthocyanins (Legua et al., 2016). Delphinidin-3,5-diglucoside is known as a major anthocyanin in pomegranate juice, and cyanidin-3,5-diglucoside, pelargonidin-3,5-diglucoside, delphinidin-3-glucoside, cyanidin-3-glucoside, pelargonidin-3-galactoside, and pelargonidin-3-glucoside are in the next ranks (Du et al., 1975). Moradi et al. examined the pomegranate peel extract (PPE) activity against adenovirus through using MTT assay with Hela cell line, and the extract was assumed to possess anti-adenovirus agents (Moradi et al., 2015). Neurath et al. investigated the inhibitory activity of pomegranate juice (PJ) against HIV-1 of the cells, which was expressing the CD4 and CXCR4 receptors as coreceptors. PJ acts directly through inhibiting the both HIV-1 and the virus receptor: CXCR4 as a coreceptor with the highest inhibitory effect. Actually, PJ inhibited the binding of CD4 to the gp120 of HIV-1 envelope. According to the evidence, it has been suggested that the PJ ingredients bound irreversibly or strongly to the binding site of CD4 on gp120 to inhibit the early stages of virus replication. It also possessed inhibitors of HIV-1 that were targeted by the gp120 virus envelope belonging to a class of antiretroviral drugs, the development of which is quite rare (Neurath et al., 2004). Altogether, the results suggest *P. granatum* as an inexpensive and safe anti-HIV-1 fruit with widely available sources.

#### Lamiaceae Family

As used in traditional and complementary medicine, the Lamiaceae family are used with outstanding antiviral activity (Cohen et al., 1964; Dimitrova et al., 1993), but there is still a lack of information regarding the antiviral effects of many plants of the Lamiaceae family (Jassim and Naji, 2003). The Lamiaceae family, like sage, basil, and thyme, has long been assessed as a rich source of unique and diverse anthocyanins. Several anthocyanin structures were isolated, consisting of cyanidin and peonidin-based pigments, such as cyanidin 3,5-diglucoside, cyanidin 3-glucoside, cyanidin based, *p*-coumaryl and malonyl acids, peonidin 3,5-diglucoside, peonidin 3-(*p*-coumarylglucoside)-5-glucoside and peonidin (aglycon) (Phippen and Simon, 1998). In their research study, Nolkemper et al. studied the activity of six species of the Lamiaceae family, including thyme (*Thymus vulgaris*), rosemary (*Rosmarinus officinalis)*, prunella (*Prunella vulgaris)*, peppermint (*Mentha x piperita)*, lemon balm (*Melissa officinalis)*, and sage (*Salvia officinalis)* against HSV. The inhibitory activities of phenolic compounds against HSV type 1 (HSV-1), type 2 (HSV-2), and HSV-1 acyclovir-resistant strain (ACV^res^) were assayed *in vitro*, which indicated a high antiviral effect against three strains of HSV time-dependently. According to the results, the extracts had efficacy in anti-HSV before adsorption stage and, in contrast, had no efficacy in the replication stage (Nolkemper et al., 2006). Therefore, the results offer a chance to use the Lamiaceae family for therapeutic application against viral diseases like HSV.

### Purified Antiviral Anthocyanin

The antiviral findings suggest that purified anthocyanin isolated from rich-anthocyanin plants may have an effective role against viral infections (Jassim and Naji, 2003). Swaminathan et al. reported the anti-InfV effects of cyanidin-3-sambubioside (C3S) isolated from black elderberry (*Sambucus nigra*) extract. From the pharmacological mechanistic point of view and according to the molecular docking of this compound, it bound with the 430-cavity adjacent to the residues of InfV neuraminidase. Since this antiviral compound bound remote from Asp 151 and Glu 119, two residues known to regulate the neuraminidase resistance, this compound was shown to have potential as a new class of antiviral drugs against the InfV without this susceptibility (Swaminathan et al., 2013). In their following study on C3S, Kannan et al. indicated that it possessed a high inhibitory activity against H274Y mutation. The inhibitory activity of C3S, oseltamivir and their mechanisms against H274Y mutant-type (MT) and wild-type (WT), were clarified using the quantum chemical methods, molecular dynamics, and molecular docking reasons of drug resistance. Oseltamivir was found to have less binding affinity with MT, while C3S possessed more position and affinity with the proteins of MT and WT. The evidence suggested that C3S had a stronger binding affinity with MT based on the energy of electrostatic interaction and was more potent against the oseltamivir-resistant virus strains (Kannan and Kolandaivel, 2018). In another study, Calland et al. attempted to discover new and novel natural compounds with potent anti-HCV activity and quantify their mode of action. Amongst the eight selected compounds, delphinidin was found to belong to the anthocyanin family and be a proper choice as a new class of flaviviridae inhibitors. The mechanism of action of this compound is that it disrupts the HCV attachment and adsorption by using the E1E2 gp of the envelope of different genotypes for HCV harboring. The time-response activity of delphinidin was also evaluated, and it was shown that its activity against HCV occurred just in the inoculation phase (Calland et al., 2015).

Vázquez-Calvo et al. studied the effect of anthocyanin, delphinidin, and cyanidin on the viral infections of flaviviruses family, including west nile virus (WNV), dengue virus (DENV), and zika virus (ZIKV). The results showed that delphinidin reduced WNV. Further assays discovered that delphinidin generally interfered with the attachment, adsorption, and entrance stages of the viral cycle and has a direct effect on the virus particles executing a virucidal activity. The same inhibitory activity of WNV variants was reported with acidic pH variation in the endosomal system for viral fusion. It was also shown that anthocyanins exhibiting antiviral activity against WNV is initiated by a virucidal activity instead of an inhibitory activity of viral fusion that was dependent on pH. Delphinidin also decreased the viral activity of DENV and ZIKV. Finally, delphinidin was reported to disrupt the viral activity of three viruses of the flaviviruses family in the cell culture (Vázquez-Calvo et al., 2017).

Swaminathan et al. assessed the activity of cyanidin, delphinidin, pelargonidin as three anthocyanins against 430-cavity of InfV neuraminidase and their binding site using molecular docking and mass spectrometry. Regardless of structural similarity, these compounds had differences not only in the position but also in the number of OH groups in the benzyl substituent connected to the chromenylium ring. The precise distinction in the binding site features was exhibited by molecular docking and mass spectrometry. Delphinidin and cyanidin, with the most number of OH substituents, bound firmly and had more inhibitory properties than pelargonidin with only one OH substituent at 4´ position. Finally, the position and number of OH groups of anthocyanins were shown to affect their antiviral activity (Swaminathan et al., 2014).

Finally, according to the importance of purified antiviral anthocyanin in combating viral diseases, clarifying the exact pharmacological mechanisms of action could pave the road for the treatment of viral infections.

### Therapeutic and Clinical Antiviral Application of Anthocyanins

In traditional medicine, plants such as elderberry (*Sambucus nigra* L.), has been used in treating the viral disease like colds and flu from past to present (Roxas and Jurenka, 2007). Some independent clinical trials proved a proper effect of elderberry extract against InfV A and B infection (Kong, 2009). In a randomized, double-blind, placebo-controlled study, elderberry extract (EBE) demonstrated a safe and therapeutic effect in the treatment of InfV A and B (Zakay-Rones et al., 2004). In another randomized, double-blind placebo-controlled clinical trial, the EBE significantly reduced the common cold symptoms, duration, and severity of cold episodes in air travelers (Tiralongo et al., 2016). Moreover, the anti-InfV activity of EBE extracts were approved in the other studies (Zakay-Rones et al., 1995; Konlee, 1998; Ulbricht et al., 2014). The efficacy of immune-modulating beverage, which is rich in polyphenols and anthocyanins on common cold and the associated symptoms was also investigated. The results showed that the group taking the verum drink exhibited a faster decrease in symptoms and sooner complaint-free compared to control group. Also, several findings revealed that the physical examination was remarkably amended (Schütz et al., 2010). *Hippophaë rhamnoides* L. (sea buckthorn) berries indicated an immunomodulatory activity and positive effects on health (Yang et al., 1999; Johansson et al., 2000; Eccleston et al., 2002; Geetha et al., 2002; Yang and Kallio, 2002; Dorhoi et al., 2006). In a double-blind, randomized, placebo-controlled trial, the antiviral effect of sea buckthorn berries was evaluated. The results showed no differences between the number or duration and prevention of common cold in receiving sea buckthorn berries group and placebo groups, however, resulted in a decrease in serum C-reactive protein (CRP), a marker of inflammation (Larmo et al., 2008).

Several other studies also showed the positive effects of plants containing anthocyanins on the number, duration, and symptoms of viral disease, especially common cold and flu (the studies are shown in **Table 3**).

**Table 3:** Human clinical trial investigations on the effects of some plant containing anthocyanins in the treatments of viral infections.

Study	Treatment	Dosage	Results
(Kong, 2009)	InfV	175 mg of the proprietary elderberry extract qid for 2 days	28% of patients were void of all symptoms, 60% showed relief from some symptoms, and had only one or two mild symptoms
(Zakay-Rones et al., 2004)	InfV A and B	15 mL of elderberry or placebo syrup qid for 5 days	Symptoms were relieved on average 4 days earlier and lessen the use of rescue medication
(Tiralongo et al., 2016)	Common cold	elderberry capsules (300 mg) or placebo bid from 10 to 2 days before travel and tid from 1 day before leaving home until 4/5 days after arriving at the destination	No difference in cold episodes, lessen in duration of cold episode days and the average symptom score.
(Zakay-Rones et al., 1995)	InfV	children received bid, and adults qid for 3 days of Sambucol^®^ as a standardized elderberry extract	Improvement of the symptoms, including fever, within 2 days. A complete cure was achieved within 2 to 3 days.
(Schütz et al., 2010)	Common cold	The 250 mL of the verum drink contained green tea, grape peel grape seed, shiitake mushroom extract as well as vitamin C, bid for 10 days.	Faster decrease in symptoms and sooner complaint free and also several findings revealed that the physical examination was remarkably amended
(Larmo et al., 2008)	Common cold	The daily dose of sea buckthorn product contained 16.7 mg of flavonol glycosides	No prevention on common cold but reduction effect on CRP, a marker of inflammation

InfV, Influenza viruses; Bid, Two times a day; Qid, Four times a day; CRP, C-reactive protein.

### The Limitations With Anthocyanins: How to Overcome?

The daily use of anthocyanins in the USA is estimated about 200 mg/day (Wang and Stoner, 2008). Although anthocyanins are ordinarily consumed in the guise of a meal, there is limited information about their bioavailability and pharmacokinetic. To comprehend the effectiveness of anthocyanins, determining the features of anthocyanin at the molecular level, their absorption (serum level), as well as urine concentration of anthocyanin red pigments after oral administration, is critical (Lucioli, 2012). Following such intakes, the plasma concentrations of anthocyanins were found to be very low. Recent studies have revealed a rapid absorption followed by a quick metabolization and excretion of anthocyanins into urine and bile as methylated, glucuronidated or intact glycosides derivatives. Some clinical studies have shown that anthocyanins are absorbed with poor efficiency (Manach et al., 2005); however, an *ex vivo* study indicated their efficient absorption (Talavera et al., 2003).

In general, the *in vivo* bioavailability of anthocyanins was found to be about 0.26–1.8% (Felgines et al., 2002; Felgines et al., 2003; Ichiyanagi et al., 2006; Matsumoto et al., 2006; Borges et al., 2007; Marczylo et al., 2009).

Maximum concentrations of anthocyanins are achieved after 1.5 h (plasma) and 2.5 h (urine) (Manach et al., 2005). The metabolites exist in the urine up until 24 h and may save the basic structure of anthocyanin (Kay et al., 2004). The urinary excretions of anthocyanins were estimated to be less than 0.1% in humans, while some studies reported up to 5% levels of anthocyanin excretion (Lapidot et al., 1998; Felgines et al., 2003). This demonstrates the extensive metabolism of anthocyanins in the body before their urine excretion (Fang, 2014). In general, the studies showed that anthocyanins are absorbed rapidly from stomach (Passamonti et al., 2003; Talavera et al., 2003) and small intestine (Miyazawa et al., 1999), and present in both blood and urine like intact, methylated, sulfate or glucuronide conjugated forms (Wu et al., 2002; Felgines et al., 2005). Anthocyanins are degraded rapidly by the microflora of the intestine so that they might be existing in the gastrointestinal tract (GIT). Although the degraded compounds are remarkably unstable in all circumstances, in the neutral pH, they are naturally changed into aldehydes and phenolic acids (Aura et al., 2005; Keppler and Humpf, 2005). There are different ways to enhance the stability and then the bioavailability of anthocyanins. Some studies showed that consumption of anthocyanin with foods influences the absorption and excretion phase. For instance, both rats and human, phytic acid hulls nuts components, grain and seeds (Dendougui and Schwedt, 2004), lead to enhance the bioavailability of blackcurrant anthocyanins (Matsumoto et al., 2007). Anthocyanins urinary recovery from rats was enhanced by increasing of phytic acid dose, which leads to the reduction of GIT mobility, decreases the anthocyanins passage through the GIT and finally plays a part in increasing the time for the absorption phase. Phytic acid also enhanced the levels of anthocyanin in human plasma and urine(Matsumoto et al., 2007). Another method to enhance the bioavailability of anthocyanins is the use of nanoformulations like nanoparticles, nanocomplexes, nanoliposomes, and nanoemulsions (Arroyo-Maya and McClements, 2015; Li et al., 2015; He et al., 2017; Chen and Stephen Inbaraj, 2019; Ge et al., 2019).

### Nano Formulation of Anthocyanins

Nano-medicine employs biodegradable and biocompatible nanoparticles to target administration routes, pharmacokinetics, and bioavailability of the target drug. Nanoparticles are regarded as sub-micron-sized particles with good dispersion, regular spherical shape, molecular forces, and surface chemical bonds (Sanvicens and Marco, 2008; Martínez-Ballesta et al., 2018). Nano-formulations could potentiate the existing drugs with antiviral activity through the positive modulation of pharmacokinetics and enhancement of bioavailability. Additionally, nano-medicine enhances the therapeutic window for antiviral medicines *via* exclusive targeting of specific sites (Jackman et al., 2016; Lembo et al., 2018).

Anthocyanins are extremely unstable hydrophilic compounds since their phenolic hydroxyl groups are easily oxidized into quinones. Moreover, they are precipitated by external factors like temperature or pH, which all affect their antiviral activities (Wu et al., 2018).

Encapsulating the anthocyanin into nanoparticles plays a significant role in overcoming this drawback and improving their antiviral properties (Amin et al., 2017; Ahsan et al., 2018). Protein encapsulation of procyanidins could attenuate their degradation under ultraviolet and other stressful conditions. According to Liu et al., nanocrystallization of anthocyanin potentiated the antioxidant capacity of pure proanthocyanidins under stressful conditions (Liu et al., 2017). These all clarify the critical role of nanocrystallization-induced improvement in the stability of anthocyanins (Arroyo-Maya and McClements, 2015; Zhang et al., 2019).

To enhance *in vitro* sustained-release characteristics, bioavailability and stability of anthocyanins, the nanocomplexes with chitosan hydrochloride (CHC), carboxymethyl chitosan (CMC) and β-Lactoglobulin (β-Lg) were provided. The results showed that nanocomplexes encapsulation significantly improved the stability and bioavailability of anthocyanins in simulated GIT (Ge et al., 2019). In another study, to improve the physicochemical stability of anthocyanins, nanoemulsion and nanoliposome were prepared. According to the results, nanoemulsion and nanoliposome enhance the anthocyanin stability and biological activity (Chen and Stephen Inbaraj, 2019). In this line, self-assembled nanoparticles were prepared by chitosan (CH) and chondroitin sulfate (CS) biopolymers to enhance the biological activity of black rice anthocyanin. The thermogravimetric analysis also proposed that the anthocyanin/CH/CS nanoparticles displayed high thermal stability. Significant increases in apoptosis by 12.1% and 35.1% were reported with 0.05 mg/ml anthocyanin and anthocyanin/CH/CS nanoparticles in the HCT-116 cell line, specifying that the nanoparticle system caused to considerable increase in apoptosis (Liang et al., 2019). In another study anthocyanins-loaded CHC/CMC nanocomplexes exhibited excellent stability against different temperature, different concentration of ascorbic acid, varying pH or white fluorescent light (Ge et al., 2018). Another investigation evaluated the bioavailability of anthocyanins-loaded nanoparticles to overcome their poor bioavailability. Based on results, pH-sensitive polymer along with the hydrophilic polymer exhibited suitable delivery system for anthocyanins. The anthocyanins delayed release profile, found in all formulations, can improve their poor bioavailability (Dupeyrón et al., 2017). Polymer-based nanoparticles are of other approaches to enhance the bioavailability of unstable hydrophilic drugs like anthocyanins. This approach is due to the improved bioavailability and high stability. Encapsulating anthocyanins in poly lactic-co-glycolic acid (PLGA)-polyethylene glycol (PEG) nanoparticles did not ruin its natural properties and showed more potent neuroprotective properties (Amin et al., 2017). Altogether, the results suggest a practical approach to enhance the efficiency of anthocyanins in the form of nano-drug delivery systems.

In this regard, deployment of nanoparticles enables the controlled release of anthocyanins, enhances their biodistribution, protects them from degradation as a result of cellular metabolism and gastrointestinal digestion, and finally, allows them to target the specific sites affected by viruses (Martínez-Ballesta et al., 2018).

## Conclusion

Natural medicine has always been considered as an excellent source against viral infections, and their discoveries would help to improve derivatives and lead-compounds to treat or even cure viral diseases. The development of novel plant-derived antiviral agents is among the top global priorities as viral infections have high mortality rates and are not yet curable.

In the present review, we highlighted the current antiviral approaches and alternative plant-derived antiviral compounds with related pharmacological mechanisms, while tackling particular attention to anthocyanins. We also focused our attention on the need to develop nanoformulation for anthocyanins in drug delivery systems to overcome the limitation with the bioavailability of anthocyanins. The potential of anthocyanin to show its antiviral effects through binding to host cells, inhibiting viral life cycle, or stimulating host immunity, strengthens the idea that anthocyanin would be an essential brick and a potential therapeutic agent to find novel antiviral lead-compounds.

Additional studies should include the investigation of other effective and novel plant-derived antiviral lead-compounds, with synergistic effects for a more favorable treatment outcome capable of enhancing immunity and reducing the cost, toxicity, and viral resistance, as well as finding their virus-specific targets and related pharmacological mechanisms of action. Synthetic campaigns could adjust these lead-compounds to find even more efficient drugs against several viral infections. Drug delivery must also be improved by new technologies using novel nano-formulation. Nonetheless, it is crucial to confirm the effects of plant-derived lead-compound in clinical trial studies.

## Author Contributions

Conceptualization: SF and MF. Designing the structure of the paper: SF and MF. Drafting the paper: PP, SF, SA, MF, and JE. Review and editing the paper: PP, SF, MF, and JE. Revising the paper: PP, SF, MF, and JE.

## Funding

JE gratefully acknowledges funding from CONICYT (PAI/ACADEMIA No. 79160109).

## Conflict of Interest

The authors declare that the research was conducted in the absence of any commercial or financial relationships that could be construed as a potential conflict of interest.

## Abbreviations

HIV, human immunodeficiency virus; RSV, respiratory syncytial virus; NTCP, Na^+^/taurocholate co-transporting polypeptide; HBV, hepatitis B virus; HCV, hepatitis C virus; HDV, hepatitis D virus; InfV, Influenza virus; HSV-1/2, herpes simplex virus-1/2; NtRTIs, nucleotide reverse transcriptase inhibitors; NRTIs, nucleoside reverse transcriptase inhibitors; NNRTIs, non-nucleoside reverse transcriptase inhibitors; ORF, open reading frame; HAART, highly active antiretroviral therapy; NA, neuraminidase; NAIs, neuraminidase inhibitors; gp, glycoprotein; TLR-3 or 7, toll-like receptor-3 or 7; IFNγ, interferon-gamma; PD-1, programmed death 1, CTLA-4, cytotoxic T-lymphocyte antigen 4; TIM-3, T-cell immunoglobulin and mucin domain-containing molecule 3; RNAi, RNA interference; siRNAs, small interfering RNAs; DDX3, Asp-Glu-Ala-Asp (DEAD)-box polypeptide 3; NS3h, non-structural protein 3 helicase; NS5B, non-structural protein 5B; VZV, varicella-zoster virus; NF-кB, nuclear factor-kappa B; PGC-1α, peroxisome proliferator-activated receptor gamma coactivator 1-alpha, EMCv, encephalomyocarditis virus; HHV-1, human herpes virus type 1; VACV-WR, vaccinia virus strains Western Reserve; SNS, *Solanum nigrum* seeds; TA, total anthocyanins content; RV, rabies virus, HPLC-DAD, High-performance liquid chromatography-diode array detector, CV-B1, coxsackievirus B1; PV-1 poliovirus type 1; HRSV-A2, human respiratory syncytial virus A2; FCV-F9, feline calicivirus-F9; MNV-1, murine norovirus-1; FBVS, foodborne virus surrogates; RCS, black raspberry seed; C3G, cyanidin-3-glucoside; MA, *Morus alba*; MAJ, *Morus alba* juice; MAS, *Morus alba* seed; Ad36, adenovirus 36; MCP-1, monocyte chemoattractant protein‐1; CJ, cranberry juice; BT2, bacteriophage T2; BT4, bacteriophage T4; GSE, grape seed extract; AJ, apple juice; GJ, gastric juice; CS, cocoa seeds; NK, natural killer cell; LWB, lacto-wolfberry; AMFJ, *Aronia melanocarpa* fruit juice; PJ, pomegranate juice; ACV^res^, HSV-1 acyclovir-resistant strain; WNV, west nile virus; DENV, dengue virus; ZIK, Zika virus; EBE, elderberry extract; CRP, c-reactive protein; Bid, two times a day; Qid, four times a day; GIT, gastrointestinal tract; CHC, chitosan hydrochloride; CMC, carboxymethyl chitosan; β-Lg, β-Lactoglobulin; CH, chitosan; CS, chondroitin sulfate; PLGA, poly lactic-co-glycolic acid; PEG, polyethylene glycol.
